# A review of disparities in access to infertility care and treatment outcomes among Hispanic women

**DOI:** 10.1186/s12958-021-00875-1

**Published:** 2022-01-03

**Authors:** Allison S. Komorowski, Tarun Jain

**Affiliations:** Division of Reproductive Endocrinology & Infertility, Department of Obstetrics & Gynecology, Northwestern University Feinberg School of Medicine, 259 E Erie St Suite 2400, Chicago, IL 60611 USA

**Keywords:** Health disparities, Reproductive medicine, Infertility care, Access to care, Hispanic ethnicity

## Abstract

Hispanic women have lower rates of use of infertility services than non-Hispanic White women. There are many barriers that impede access to infertility care including economic, geographic, cultural, and societal factors and there are disparities in treatment outcomes. Hispanic women are less likely to seek infertility care than non-Hispanic White women and even after infertility evaluation, Hispanic women are less likely to receive treatment for their infertility. Lower use of infertility treatments among Hispanic women is unlikely to be driven solely by economic factors. There is disappointingly little data on in-vitro fertilization treatment outcomes including the population of Hispanic women, and existing data has yielded conflicting results. Incomplete and variable reporting of race data across clinics raises the potential for misclassification bias and invalid study conclusions. Addressing disparities in access to reproductive medicine in the Hispanic population will required a multifaceted approach including expanded insurance coverage, improved education for both patients and providers, and additional research on barriers to care.

## Background

Impaired fecundity affects approximately 7.4 million women in the United States, corresponding to 12.1% of reproductive-age women [1]. According to the 2019 U.S. Census Bureau population estimate, people with Hispanic/Latino ethnicity comprised 18.4% of the U.S. population. However, Hispanic women have lower rates of use of infertility services than non-Hispanic White women [2, 3]. There are many barriers that impede access to infertility care including economic, geographic, cultural and societal factors and there are disparities in treatment outcomes. To identify potential studies for inclusion, PubMed was searched for the terms “Hispanic infertility” and “Hispanic IVF outcomes”, however not all articles were included in this narrative review. The aims of this review include examining disparities in reproductive medicine affecting Hispanic women and outlining strategies to promote reproductive health equity in this population.

## Main text

### Access to care

A large national survey of over 10,000 reproductive age women by the National Center for Health Statistics in 1995 found that Hispanic women reported infertility more often than Caucasian women [4]. More recent data has showed a similar prevalence of infertility across racial groups; however, Hispanic women are underrepresented in the population receiving infertility treatment compared to the demographics of reproductive-age women in the United States [2, 5]. Evaluation of the Society of Assisted Reproductive Technology data from 1999 to 2000 from over 80,000 patients revealed that Hispanic patients comprised only 5.4% of the U.S. assisted reproductive technology (ART) population [6]. Based on data from the 2002 National Survey of Family Growth, 15% of White women between ages 25–44 years in the United States have sought medical help to get pregnant compared to only 7.6% of Hispanic women [5]. This discrepancy may be due to several barriers including lack of insurance coverage, language, geography, economic, social stigmas, religious beliefs and cultural emphasis on privacy [1]. The findings of the aforementioned study by Chandra et al. and several others mentioned below are summarized in Table 1.

**Table 1:** Summary of studies addressing access to infertility care in Hispanic patients

Authors	Year Published	Study Design	Location	Sample Size	Outcomes/Highlighted Findings	Study Limitations
Chandra et al. [5]	2005	National survey	United States	12,571	7.6% of Hispanic women ages 25-44 have sought medical help to get pregnant compared to 15% of White women	Descriptive; risk of non-sampling error; risk of recall error
Jain & Hornstein [13]	2005	Cross-sectional survey	Massachusetts	561	6.8% of the MA state population identified as Hispanic/Latino as compared to 3.9% of patients who presented for care to a large fertility center in a state with mandated insurance coverage for services (*p*=.011)	Descriptive; risk of non-sampling error; patients from single fertility center
Feinberg et al. [14]	2007	Retrospective chart review	Washington D.C.	1,457	Hispanics comprised 9% of the Department of Defense population and 4% of the ART population	Low number of Hispanic patients; specific military population
Greil et al. [8]	2011	Path analysis of telephone survey data	United States	2,162	Hispanic and Black women had higher infertility stigma scores and more ethical concerns surrounding infertility than White women	Descriptive; risk of non-sampling error
Missmer, Seifer & Jain [7]	2011	Cross-sectional survey	Illinois	743	Hispanic patients had been trying to conceive 20 months longer than White patients when presenting for care; Hispanic patients reported it was more difficult to get treatment due to race/ethnicity (OR 36, 95% CI 6.6-195)	Descriptive; patients from single fertility center
Dupree et al. [15]	2019	Retrospective chart review	Michigan	18,282	Following implementation of employer-sponsored IVF coverage, the absolute rate of increase in IVF among Hispanic women was 27.5% (*p*=.25) compared to an increase of 64.9% among White women (*p*<.001)	Data from single employer; no control group
Galic et al. [9, 10]	2021	Cross-sectional survey	Illinois	1,460	Hispanic patients traveled twice as far for treatment (*p*=.01) and were more likely to report race/ethnicity treatment barriers than White patients (*p*=.01); Hispanic patients were more concerned about side effects of treatment (*p*<.05) and to worry about violating religious beliefs than White patients (OR 2.3, 95% CI 0.8-6.5)	Descriptive; patients from single fertility center

A cross-sectional survey of Midwestern infertility patients conducted from 2004 to 2005 attempted to identify cultural factors contributing to disparities [7]. Hispanic and African-American women had been attempting to conceive for 20 months longer than White women and were significantly more likely to be nulliparous when presenting for infertility care. Hispanic and African-American women were also significantly more likely to report difficulties finding a physician with whom they felt comfortable, getting appointments with a physician, taking time off work for their appointment, and paying for treatment. Importantly, Hispanic and African-American women reported it was more difficult to get treatment because of their race or ethnicity. Hispanic women were more likely than White women to report significant concerns or worries regarding needing to consider adoption as an alternative to treatment first, friends or family finding out about treatment, using science or technology to conceive, short and long-term side effects, having a miscarriage, having an ectopic pregnancy, or having triplets or greater. Similarly, a telephone survey of American women examining factors involved in reproductive choices and infertility found that Hispanic and Black women had higher infertility stigma scores and more ethical concerns surrounding infertility than White women [8].

More recently, a cross-sectional survey of 1460 women seeking fertility care at an academic center in Illinois, a state with mandated insurance coverage for fertility testing and treatment, assessed racial and socioeconomic characteristics and access to care [9]. Hispanic women in this population traveled twice as far as White and Black women. Additionally, Hispanic and Black women were twice as likely to report their income level and weight as barriers to treatment compared to White and Asian women [9]. Hispanic patients were significantly more likely than White patients to report being very/extremely worried about side effects of treatment, miscarriage, ectopic pregnancy and birth defects [10]. Compared to White patients, Hispanic patients were twice as likely to report being very/extremely worried about using science and technology to conceive and were more likely to report worry about violating religious beliefs [10]. Additionally, 81% of Hispanic women reported a belief that emotional stress can reduce success of fertility treatment compared to 67% of White women, a statistically significant difference [11]. Hispanic women were also significantly more likely to report that emotional stress can cause a miscarriage than White women [11]. Regarding genetic carrier screening, Hispanic women were significantly less likely to have had this testing compared to White women [12]. In this study, only 49.4% of Hispanic infertility patients had genetic carrier screening performed [12]. Hispanic patients were less likely than White and Asian patients to agree with the statement that using left-over embryos for research should be allowed [12].

Financial barriers alone are unlikely to be the major barrier for access to care in this population. In 2003, a survey of Massachusetts women was conducted as mandated and comprehensive insurance for infertility services became available [13]. Based on Massachusetts Census data in 2000, 6.8% of the state population identified as Hispanic/Latino ethnicity as compared to 3.9% of patients who presented for care at a large infertility clinic; this difference reached statistical significance [13]. Additional information comes from the study of Feinberg et al. on use of ART in the military health care system, which can be thought of as an equal-access-to-care model [14]. Retrospective study of 1457 patients revealed that in the lower cost setting, use of ART among Hispanic patients did not increase; while Hispanics comprised 9% of the Department of Defense population, they comprised only 4% of the ART population [14]. Compared to the general population, education and language barriers were considered less likely in the Department of Defense population given only 6.5% of Hispanic members had not completed high school and at least one member of the Hispanic couple was English speaking given active duty service member requirements [14]. Additionally, financial barriers are less likely in the Department of Defense population given lower cost ART services available [14]. Similarly, Dupree et al. assessed changes in rate of use of in-vitro fertilization (IVF) among over 18,000 health plan enrollees after a large employer provided IVF coverage [15]. The absolute rate increase in IVF use among Hispanic women was the lowest of the racial groups studied and at 27.5% which was not statistically significant, while the absolute rate significantly increased among White women at 64.9% [15]. The findings of these studies demonstrate that lower use of ART among Hispanic women is unlikely to be driven solely by economic factors and that social and cultural factors may be contributing [14].

### Infertility treatment

Hispanic women are less likely to seek infertility care than non-Hispanic White women; moreover, even after infertility evaluation, Hispanic women are less likely to receive treatment for their infertility [16]. Infertility has many potential etiologies including, but not limited to, ovulatory dysfunction, tubal factors, uterine factors, hypothalamic, genetic, and male factors. Hispanic women are disproportionately affected by obesity, with 43% of Hispanic women affected in the United States as of 2017–2018 [17]. Women with obesity have higher rates of ovulatory dysfunction, increased likelihood of infertility and worse outcomes when treated with ART [18]. Hispanic women also have higher rates of tubal factor infertility, which requires more invasive treatment with IVF [19–21].

Regarding IVF treatment outcomes, there is disappointingly little data including the population of Hispanic women, and existing data has yielded conflicting results. A large IVF outcomes study including Hispanic women was conducted by Fujimoto et al. in 2010, which reviewed the Society for Assisted Reproductive Technology Clinic Outcome Reporting System (SART-CORS) national database from 2004 to 2006 [21]. This study and several others mentioned below regarding disparities in infertility treatment outcomes are summarized in Table 2. The SART-CORS database includes data from more than 90% of all reporting clinics, comprising more than 90% of ART cycles performed in the United States. All ART cycles using non-donor oocytes and partner semen were reviewed, which included 139,027 IVF cycles with fresh embryo transfers (8969 of which were in Hispanic women); compared to White women, the odds of live birth were significantly reduced in Asian, Black and Hispanic women [21]. Hispanic women were 13% less likely than White women to have a live birth as their pregnancy outcome [21]. Moreover, Hispanic women were significantly more likely than White women to have preterm deliveries and low birth weight infants [21]. More recently, Kotlyar et al. reviewed the SART-CORS database ART outcomes from 2014 to 2016 and found significantly lower cumulative live birth rates in Hispanic women compared to non-Hispanic white women; odds ratio for live birth in Hispanic women without prior ART was 0.82 (95% CI 0.73–0.91, *p* < .001) and with prior ART was 0.87 (95% CI 0.77–0.99, *p* = .031) [22]. The potential reasons for worse outcomes in Hispanic women are unknown and could range from differences in genetics, ovulatory function, male factor, diet and environment.

**Table 2:** Summary of studies assessing infertility treatment outcomes in Hispanic patients

Authors	Year Published	Study Design	Location	Sample Size	Outcomes/Highlighted Findings	Study Limitations
Grainger et al. [6]	2004	Retrospective cohort study of SART 1999-2000	United States	68,512	No difference in clinical intrauterine gestation or live birth rates for Hispanic women compared to White women undergoing ART treatment	Fewer cycles in Hispanic women than more recent studies; variability in clinic reporting of race/ethnicity data
Feinberg et al. [14]	2007	Retrospective chart review	Washington D.C.	1,457	No significant difference between Hispanic and White ART patients for outcomes of clinical pregnancy rates, live birth rates, implantation rates, or spontaneous abortion rates (all *p*-values non-significant)	Low number of Hispanic patients; specific military population
Fujimoto et al. [21]	2010	Retrospective cohort study of SART-CORS 2004-2006	United States	139,027	Hispanic women were 13% less likely than White women to have a live birth as their pregnancy outcome from ART (*p*=.005); Hispanic women were more likely to have preterm deliveries (*p*=.001) and low birth weight infants after ART treatment than White women (*p*<.0001)	Variability in clinic reporting of race/ethnicity data; lack of BMI and socioeconomic status data
Shuler et al. [20]	2011	Retrospective chart review	Texas	435	No differences were seen between Hispanic patients and non-Hispanic White patients for clinical intrauterine gestation rates or live birth rates (*p*=.59 and *p*=.27, respectively)	Patients from single fertility center; lack of socioeconomic status data
McQueen et al. [23]	2015	Retrospective chart review	Illinois	4,045	Hispanic women were more likely to have their IVF cycle cancelled than non-Hispanic White women (*p*=.001); Hispanic women had similar pregnancy outcomes to White women with similar clinical pregnancy rates (*p*=.50) spontaneous abortion rates (*p*=.97) and live birth rates (*p*=.48)	Patients from single fertility center; lack of ability to control for confounders including embryo quality and comorbidities; lack of socioeconomic status data
Kotlyar, Simsek & Seifer [22]	2021	Retrospective cohort study of SART-CORS 2014-2016	United States	148,572	Hispanic women had lower cumulative live birth rates compared to non-Hispanic White women; odds ratio for live birth in Hispanic women without prior ART was 0.82 (95% CI 0.73-0.91, *p*<.001) and with prior ART was 0.87 (95% CI 0.77-0.99, *p*=.031)	39% of cycles lacked race/ethnicity data; lack of socioeconomic status data

Other studies have not identified differences in IVF outcomes for Hispanic patients. Grainger et al. reviewed racial disparities in ART outcomes utilizing the SART database from 1999 to 2000 and found no difference in clinical intrauterine gestation rates or live birth rates in Hispanic women compared to White women [6]. However, during this time period, there were fewer cycles of Hispanic patients (4338) as compared to the more recent SART studies described above. In the retrospective study by Feinberg et al. previously described of 1457 patients undergoing ART in the military health care system, there was no significant difference between Hispanic and White patients for outcomes of clinical pregnancy rates, live birth rates, implantation rates, or spontaneous abortion rates [14]. Shuler et al. reviewed IVF cycle data from a fertility center in Texas from 1998 to 2008 which included 134 Hispanic women and 301 non-Hispanic White women and found no significant differences in clinical pregnancy rates or live birth rates [20]. More recently, McQueen et al. studied 4045 women undergoing their first fresh non-donor IVF cycle at a large private practice in Chicago between 2010 and 2012 [23]. Hispanic women were more likely to have their IVF cycle cancelled than non-Hispanic White women. However, Hispanic women had similar pregnancy outcomes to White women with similar clinical pregnancy rates (34% vs 36%, *P* = .50), spontaneous abortion rates (15.3% vs 14.6%, *P* = .97) and live birth rates (28.5% vs 30.7%, *P* = .48).

There is substantial need for additional research regarding ART outcomes in the Hispanic population given the paucity of data and lack of consistent findings. A major barrier to improved understanding of disparities is that race and ethnicity are not consistently reported by providers in the SART-CORS database; fewer than 65% of SART reported cycles include this information [24]. Incomplete and variable reporting of race data across clinics raises the potential for misclassification bias and invalid study conclusions [25].

### Strategies for improvement

There are several potential strategies to improve access to infertility care, social stigmas surrounding infertility and ART outcomes in Hispanic women. Similar strategies have been proposed by others in approaching the disparities in access and outcomes for African-American women [25–28]. Improved insurance coverage for infertility evaluation and treatment may encourage more patients to seek care and to eliminate some financial barriers. Additionally, improved access to primary care and general gynecologic care is crucial as many causes of infertility could be prevented if caught early. For example, prevention and early treatment of obesity and sexually transmitted infections could lead to reductions in infertility and tubal factor infertility rates, respectively. Hispanic women in America have historically had lower rates of cervical cancer screening; this has recently seen improvements with expansion of Medicaid coverage [29]. Since general gynecologists often refer patients to Reproductive Endocrinology and Infertility (REI) specialists for infertility evaluation and treatment, access to general gynecology care is critical to improving access to infertility care. However, as previously described, Hispanic women may be less likely to seek infertility care even with adequate insurance coverage [15]. Future research efforts should focus on better understanding access to care and ART outcomes in the Hispanic population and to do so, more complete reporting of race data across clinics will be needed.

Educational programming for patients addressing the causes for infertility and destigmatizing infertility may also improve both access to care and utilization of ART treatments. As previously described, Hispanic women may perceive more social stigma surrounding infertility and may have more ethical and religious concerns about ART treatment. Serou and Quintero hypothesized that a major factor in reduced ART utilization rates among Hispanic patients is the lack of fertility information in commonly used Spanish media and social networks [30]. Educational materials for patients should emphasize that infertility is a medical condition, that evaluation and treatment are available, and that IVF is not the only treatment option.

Improved awareness among general gynecologists and infertility specialists of ovulatory dysfunction in women with obesity and polycystic ovary syndrome (PCOS) and tubal factor infertility will aid earlier identification of women at-risk for infertility. As highlighted by a recent review on disparities for African-Americans in reproductive medicine, there is evidence from survey data that obstetrics and gynecology physicians-in-training have inadequate knowledge surrounding PCOS; only 10% of trainees were able to successfully identify the 3 Rotterdam criteria used in the diagnosis of PCOS [25, 31]. Additional survey data of residents in obstetrics and gynecology demonstrates significant gaps in fertility knowledge and that trainees overestimated the per cycle success rates of ART [32]. It is imperative that residency programs improve their training in REI so that physicians graduating as obstetrician-gynecologists have adequate fertility knowledge to counsel and refer patients.

Physician education on implicit bias, cultural competency and providing care to multicultural populations could serve to improve the patient experience. Physicians who care for diverse populations of patients may provide different levels of care to different racial and ethnic groups; evidence exists that physicians with higher implicit bias scores were more likely to treat white patients than black patients with thrombolysis for myocardial infarction [33]. Providers may be unaware of their implicit biases and the role they play in perpetuating health disparities in their fields [34]. Patient satisfaction, adherence to treatment and health outcomes can be optimized by effective patient-physician communication; however, patients of minority background may have worse satisfaction with physician communication than White patients [35, 36]. Providers who receive education on the barriers in access to care and common fears regarding ethical and religious concerns for ART treatment may be able more effectively communicate and better advise minority patients on treatment options.

Finally, there remains significant lack in minority representation in the physician population, which is not reflective of the general United States population. The majority of active physicians identify as White (56.2%), followed by Asian (17.1%) with much lower proportions identifying as Hispanic (5.8%) and Black or African-American (5.0%) [37]. Within obstetrics and gynecology, minorities remain underrepresented with 9.6% identifying as Black or African-American and only 6.7% as Hispanic [37]. Within REI, a survey of members of the professional society for specialty-trained physicians practicing reproductive medicine found that most REI physicians identified as Caucasian (76%) with only 6% identifying as Hispanic and 3% as African-American [38]. To improve these disparities, residency programs should seek to actively recruit underrepresented minority applicants and consider offering second visits to these applicants [37]. Institutions should address diversity in hiring practices and seek to advance women and underrepresented minorities into leadership roles [39].

## Conclusions

Hispanic women receive disproportionately lower rates of infertility treatment and may experience worse IVF outcomes than non-Hispanic White women. Addressing disparities in access to infertility care in the Hispanic population will required a multifaceted approach including expanded insurance coverage, improved education for both patients and providers, and additional research on barriers to care.

## Data Availability

Not applicable.

## Abbreviations

ART: Assisted reproductive technology
IVF: In-vitro fertilization
SART-CORS: Society for Assisted Reproductive Technology Clinic Outcome Reporting System
REI: Reproductive Endocrinology and Infertility
PCOS: Polycystic ovary syndrome
